# Cuffed endotracheal tube as a cardiopulmonary bypass venous return cannula

**DOI:** 10.1016/j.xjtc.2022.02.026

**Published:** 2022-02-22

**Authors:** Klaudiusz Stoklosa, Juan R. Contreras, Robert J. Cusimano

**Affiliations:** a Faculty of Medicine, University of Toronto, Toronto, Ontario, Canada; b Division of Cardiac Surgery, Peter Munk Cardiac Centre, University Health Network - Toronto General Hospital, University of Toronto, Toronto, Ontario, Canada; c Department of Surgery, Universidad de la Frontera, Temuco, Chile

**Figure:**
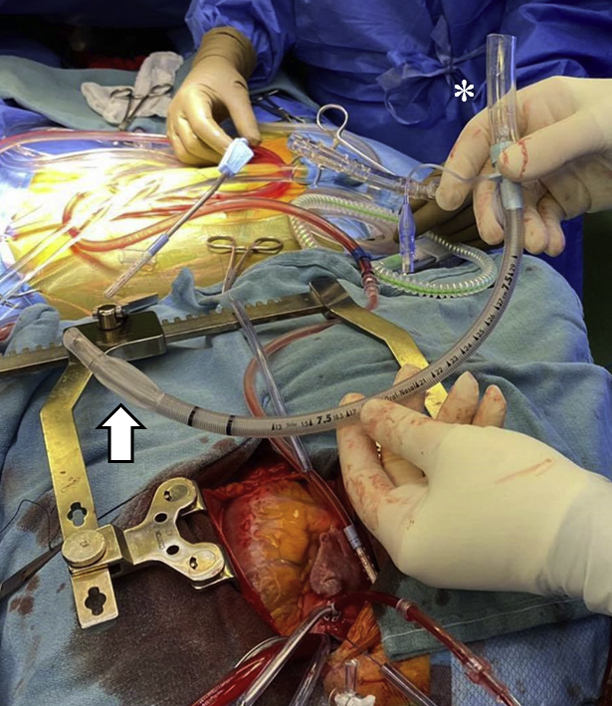
Cuffed, endotracheal tube as cardiopulmonary bypass venous return cannula.

**Central Message**

A cuffed endotracheal tube was used as a cardiopulmonary bypass IVC return cannula in a patient with an in situ IVC-RA stent and no femoral vein access.

A 49-year-old White man presented with congestive heart failure from an iatrogenic aorto-right atrial (RA) fistula secondary to an inferior vena cava (IVC) stent placed 1 year previously. The patient suffered from antiphospholipid antibody syndrome for 12 years. During this time, he developed Budd-Chiari syndrome secondary to IVC thrombosis and was considered for liver transplantation. IVC stenting into the RA resolved his liver concerns. Unfortunately, although his liver issues were resolved, he subsequently developed heart failure and both an aorto-right atrial fistula and an atrial septal defect were discovered, possibly related to the IVC stent (Figure 1). An interventional approach for repair was not feasible.

**Figure 1:**
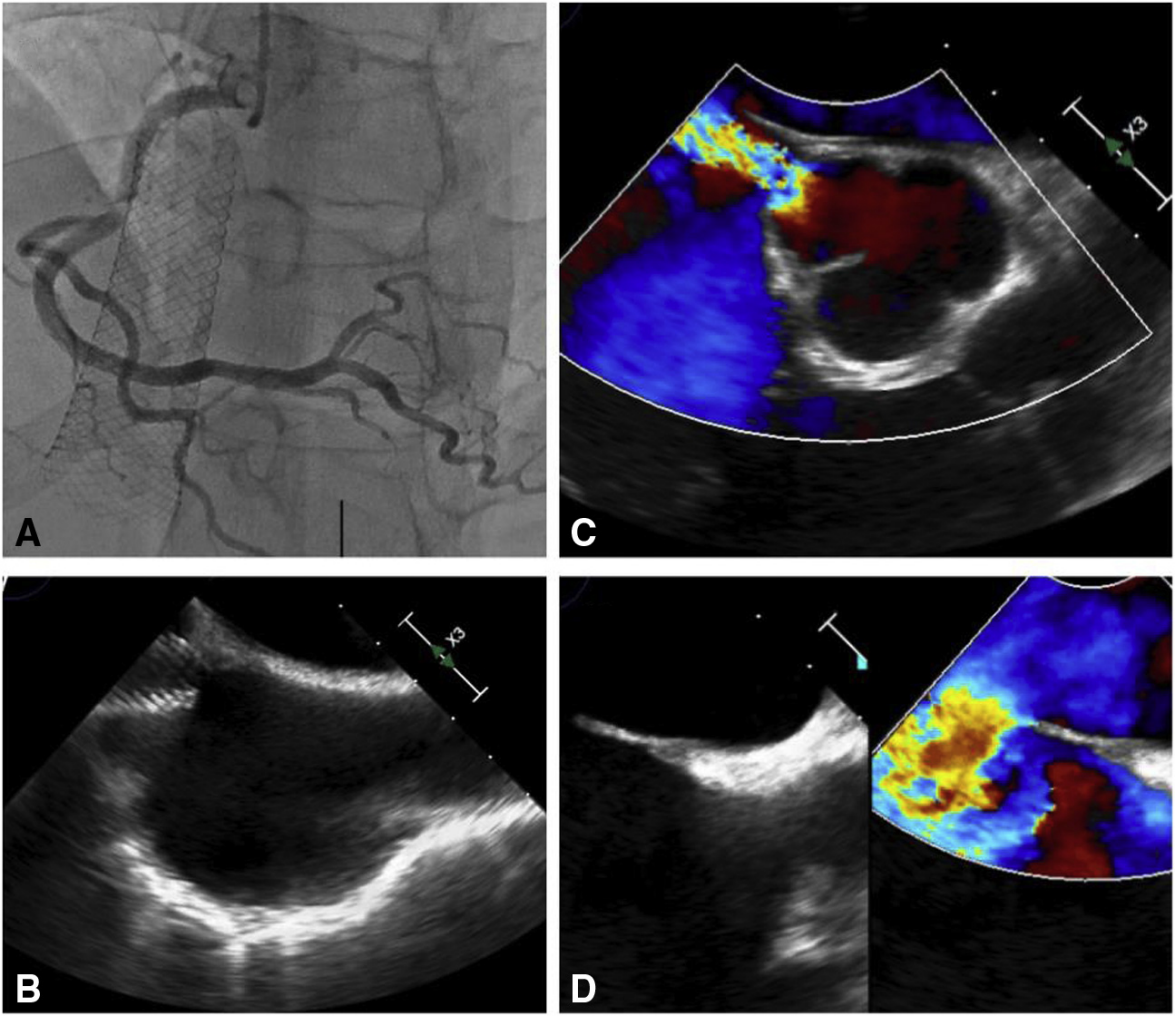
A and B, Inferior vena cava stent and relation in the right atrium. C, Aorto-right atrial fistula, connection in the noncoronary sinus. D, Large secundum atrial septal defect.

The patient was scheduled for surgical repair. Due to the rigid IVC-RA stent, conventional IVC cannulation and snaring was not possible. Preoperative peripheral venous Doppler demonstrated subtotally occlusive, bilateral common femoral, femoral, and popliteal vein thromboses, precluding femoral cannulation for venous return. Instead, a cuffed endotracheal tube (ETT) was used. When inflated, it provided a seal against the IVC stent. Intraoperatively, a size 7.0 armored, cuffed ETT (Smiths Medical International) was assembled (Figure 2). Internal and external ETT diameters were 7.0 mm and 9.6 mm, respectively. A conventional IVC cannulation technique was used, and the uninflated ETT was advanced until it was felt the cuffed portion was at the level of the intrahepatic IVC stent. The ETT cuff was inflated and the cardiopulmonary bypass (CPB) circuit was connected (Figure 3; Video 1). The atrial septal defect was subsequently closed using a pericardial patch and aorta-RA fistula closed using pledgeted stitches. The total crossclamp time and pump time were 29 minutes and 54 minutes, respectively. After CPB discontinuation, the cuffed ETT was removed from the IVC without injury or stenosis in the IVC stent (Video 2). The patient was discharged home on the eighth postoperative day. The patient has provided expressed consent to publish this case report.

**Figure 2:**
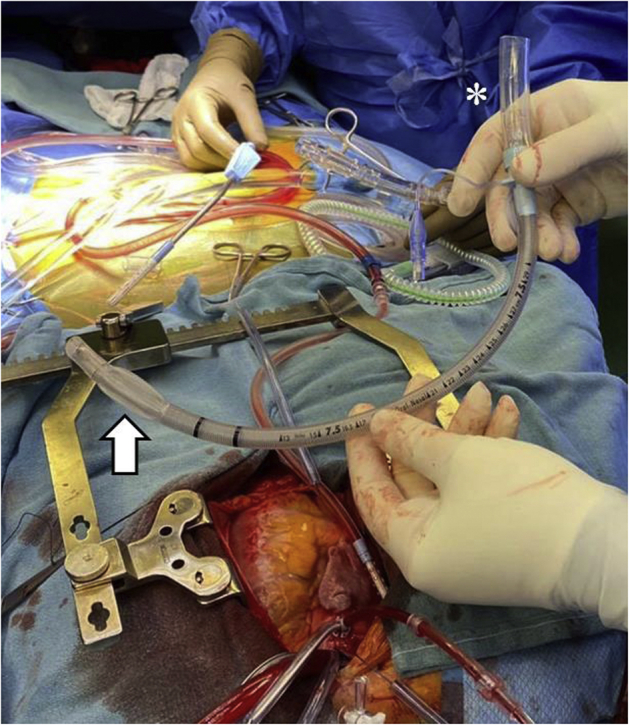
Size 7.0 armored, cuffed endotracheal tube connected to cardiopulmonary bypass cannula tubing (*asterisk*). The cuff is deflated (*arrow*).

**Figure 3:**
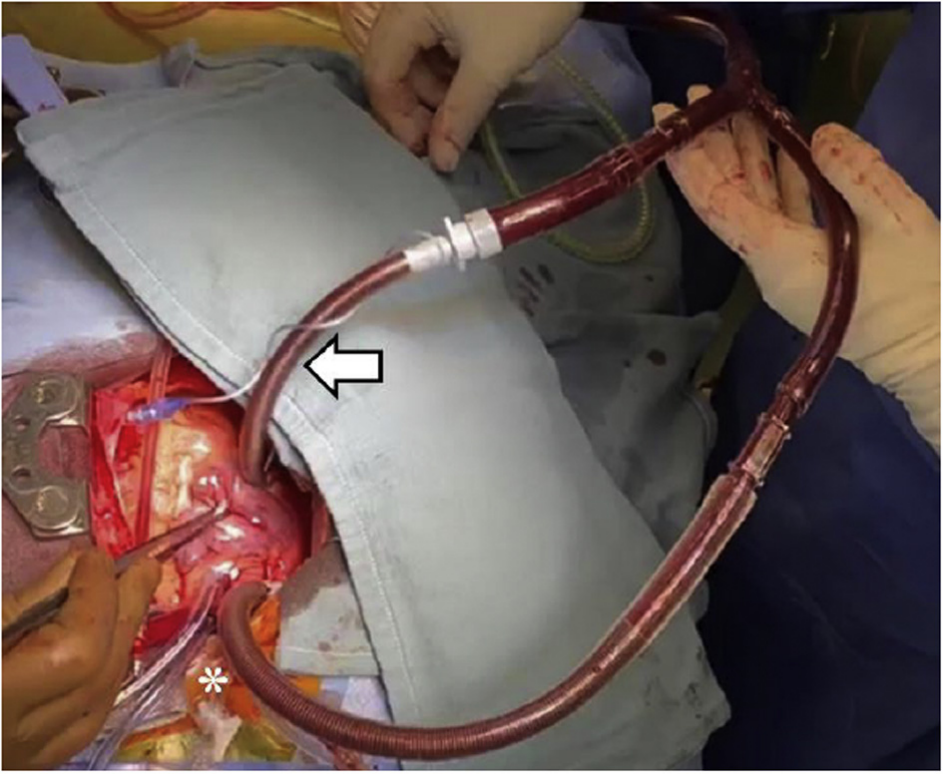
Intraoperative cardiopulmonary circuit. Superior vena cava cannula (*asterisk*) is joined with the inflated, armored, cuffed endotracheal tube inferior vena cava return cannula (*arrow*) for venous return.

## Discussion

It is well known that dry operative fields expedite cardiac procedures. Although cardiac surgeons have benefited from CPB for several decades, advancements in venous cannula designs have been limited. To our knowledge, no cannulas exist that would allow adequate drainage from a stented vena cava.

We present a case report of a 49-year-old male patient with an iatrogenic aorto-right atrial fistula following an IVC stent insertion to treat hepatic IVC thrombosis. Conventional IVC cannulation through the right atrium could not be effectively achieved, as an incomplete seal from the size mismatch between the inner surface of the stent and outer surface of the cannula would preclude safe bypass. Femoral vein cannulation was not possible given the extensive, bilateral occlusion of the common femoral veins. The surgical team considered simultaneously introducing a drop sucker and a pump sucker, both at full suction, but this was also considered inefficient. Performing the operation under deep hypothermic circulatory arrest was also not ideal, given the cerebral, cardiovascular, renal, and coagulative complications associated with hypothermic arrest.^1^

The use of cuffed ETTs during CPB operations has previously been described as left ventricular vents and for temporarily sealing ruptured right atrial walls during cannulation.^2^^,^^3^ We used the ETT cuff's ability to expand and create a near-perfect seal against the stent, allowing for successful IVC cannulation. The clear operative field simplified a complex operation, thereby minimizing crossclamp time. This technique could also be considered for superior vena cava stents.

This cannulation technique is not without its risks and considerations. The surgical team must decide on an ETT that is narrower than the stent's intraluminal diameter. This could be facilitated by preoperative imaging, but the surgeon must be ready for intraoperative discrepancies. Moreover, the cuff is at risk of rupture from an exposed or sharp stent wire edge. Finally, surgeons must be cognizant of the depth of insertion of the tube, as prolonged obstruction of hepatic veins by a deeply placed cuff may be deleterious.^4^

## Conclusions

A thorough surgical plan for a bloodless operative field is instrumental for minimizing operative time and maximizing chances of success. This report describes the successful use of a cuffed ETT as an IVC return cannula in a patient with a large IVC stent in situ and no femoral vein access.

## Supplementary Data

Video 1 Intraoperative CPB circuit from the surgeon's view. Superior vena cava cannula is joined with the inflated, armored, cuffed endotracheal tube inferior vena cava return cannula for venous return. Video available at: https://www.jtcvs.org/article/S2666-2507(22)00137-7/fulltext.

Video 2 After CPB discontinuation, the cuffed endotracheal tube was removed from the IVC without injury or stenosis in the IVC stent. Video available at: https://www.jtcvs.org/article/S2666-2507(22)00137-7/fulltext.
