# Supplementary figures and images for: Cuffed endotracheal tube as a cardiopulmonary bypass venous return cannula
Source: JTCVS Tech. 2022 Feb 22;13:89–91. doi: 10.1016/j.xjtc.2022.02.026 (PMC9196316; doi:10.1016/j.xjtc.2022.02.026)

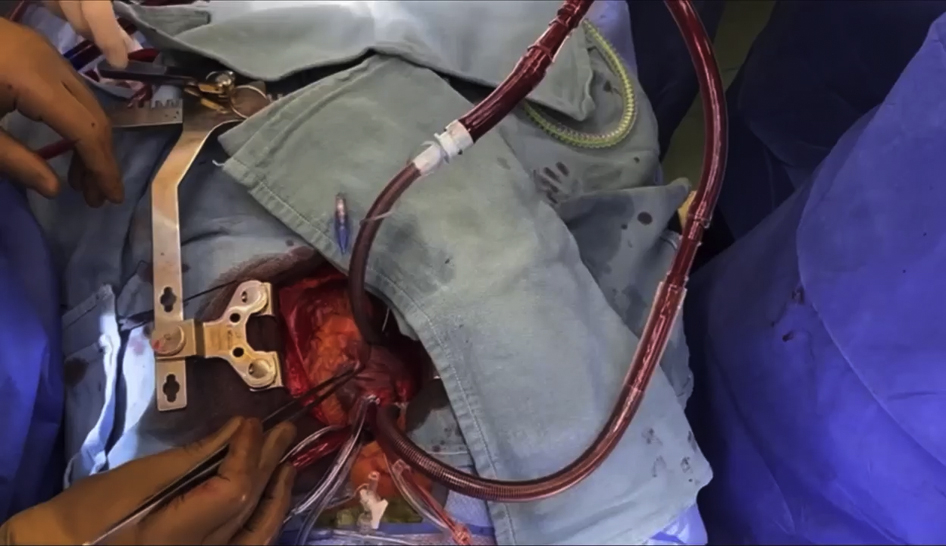

Supplement: Video 1 — Intraoperative CPB circuit from the surgeon's view. Superior vena cava cannula is joined with the inflated, armored, cuffed endotracheal tube inferior vena cava return cannula for venous return. Video available at: https://www.jtcvs.org/article/S2666-2507(22)00137-7/fulltext. [file fx2.jpg]

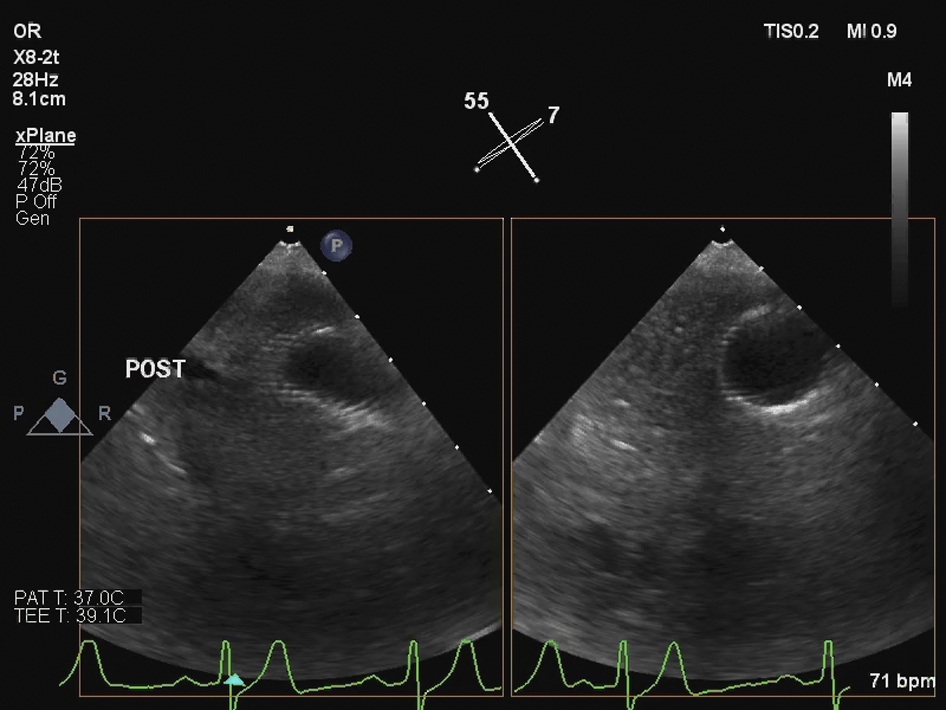

Supplement: Video 2 — After CPB discontinuation, the cuffed endotracheal tube was removed from the IVC without injury or stenosis in the IVC stent. Video available at: https://www.jtcvs.org/article/S2666-2507(22)00137-7/fulltext. [file fx3.jpg]
